# Ghrelin accelerates the growth and osteogenic differentiation of rabbit mesenchymal stem cells through the ERK1/2 pathway

**DOI:** 10.1186/s12896-015-0176-2

**Published:** 2015-06-09

**Authors:** Nan Ye, Dianming Jiang

**Affiliations:** Department of Orthopedic, The First Affiliated Hospital of Chongqing Medical University, Yuzhong District Yueyuan Road No 1, Chongqing, China; Dpartment of Cervical Surgery, The second Affiliated Hospital of Inner Mongolia Medical University, Muslims camp Square Road No 1, Hohhot, China

**Keywords:** rBMSC, MAPK, Ghrelin, Osteogenic differentiation

## Abstract

**Background:**

Mesenchymal stem cells (MSCs) can differentiate into chondroblasts, adipocytes, or osteoblasts under appropriate stimulation. Ghrelin, an endogenous ligand for the growth hormone secretagogue receptor (GHSR), stimulates growth hormone (GH) secretion, and has both orexigenic and adipogenic effects. This study sought to understand the potential involvement of members of MAPK serine/threonine kinases in the ghrelin-induced growth of rabbit MSCs ( rBMSC).

**Methods:**

We applied various concentrations of ghrelin to cultured rBMSC and observed the growth rate of the cells by MTT, changes in the phosphorylation state of ERK1/2, JNK and p38, and the expression levels of ALP, Runx2, and Osterix by wetern blot.

**Results:**

We found that the growth and osteogenic differentiation of ghrelin-treated rBMSC are promoted primarily by phosphorylated ERK1/2, and that this phosphorylation, as well p38 phosphorylation, is mediated by GHSR.

**Conclusions:**

Our study suggests that ghrelin promotes the growth and osteogenic differentiation of rBMSC primarily through the ERK1/2 pathway.

## Background

Mesenchymal stem cells (MSCs) are regarded as one of the promising candidates for cell therapy by either autologous or allogeneic transplantation [1]. MSCs can differentiate into a variety of cell types, including osteocytes, chondrocytes, adipocytes, cardiomyocytes, neurons, and endothelial cells [2–4]. Transplanted MSCs can mobilize and become integrated within the host to replace pathological or damaged tissue [5]. In addition, MSCs can modulate immune responses and inflammation, providing growth factors or cytokines that can prevent cells from undergoing apoptosis in certain environments, notably in the central nervous system [6]. During the last two decades, an increasing number of studies have proved the therapeutic potential of MSCs in the treatment of neurodegenerative diseases, spinal cord and brain injuries, cardiovascular diseases, diabetes mellitus, and diseases of the skeleton [7–9]. Basic research on MSCs in certain fields has led to the initiation of clinical trials worldwide. To provide therapeutic benefits and further understand the mechanisms responsible for them, large numbers of cells are needed [10, 11]. Thus, it is important to find efficient, economical and scalable methods to generate large numbers of MSCs without altering their multipotency [12].

Ghrelin, an endogenous ligand for the growth hormone secretagogue receptor (GHSR), is a 28-amino acid peptide produced from a 117-amino acid preprohormone. The mature form of ghrelin may undergo several post-translational modifications, including the addition of a fatty acid chain (n-octanoic acid) to the serine residue at position 3 [13]. Ghrelin has been shown to stimulate growth hormone (GH) secretion, to have both orexigenic and adipogenic effects [14], and to influence the metabolism of both glucose and lipids. Most ghrelin is produced in the stomach, by a distinct group of endocrine cells located within the gastric oxyntic mucosa [15, 16], with smaller amounts produced by other organs. Small amounts of ghrelin have also been observed elsewhere in the gastrointestinal tract and in the pancreas.

The MAPKs are members of a family of serine/threonine kinases that play an essential role in transmitting the activation of cell-surface receptors to effect downstream changes in transcriptional programs. They are expressed ubiquitously and are involved in the regulation of a wide variety of critical cellular functions, including proliferation, differentiation, migration and apoptosis [17]. In humans, there are at least 11 members of the MAPK superfamily, which can be divided into six distinct subgroups based on sequence similarity: ERK1 and ERK2; JNK1, JNK2 and JNK3; and the p38 MAPKs. Each group of MAPKs is activated by a distinct kinase cascade in which a MAP3K (or MEKK) phosphorylates and activates a downstream dual-specificity MAP2K (or MEK), which in turn stimulates MAPK activity through dual phosphorylation on threonine and tyrosine residues within a conserved tripeptide motif (Thr-X-Tyr). Phosphorylation of these threonine and tyrosine residues results in a conformational change that increases the accessibility of the active site and enhances catalysis [18–20]. ERKs are activated in response to various cytokines and growth factors and mediate primarily mitogenic and anti-apoptotic signals [21].

While several studies have focused on ghrelin’s effect on the growth of neural stem cells and embryonic stem cells, few have examined its influence on MSCs. Here, we used ghrelin to investigate the molecular mechanisms underlying rabbit MSC differentiation to osteoblasts and to enhance the osteogenic potential of rabbit MSCs. Our data revealed that ghrelin triggers osteogenic differentiation of rabbit bone marrow-derived mesenchymal stromal cells through ERK1/2 signaling pathways.

## Results

### Identification of rBMSC

**Fig. 1:**
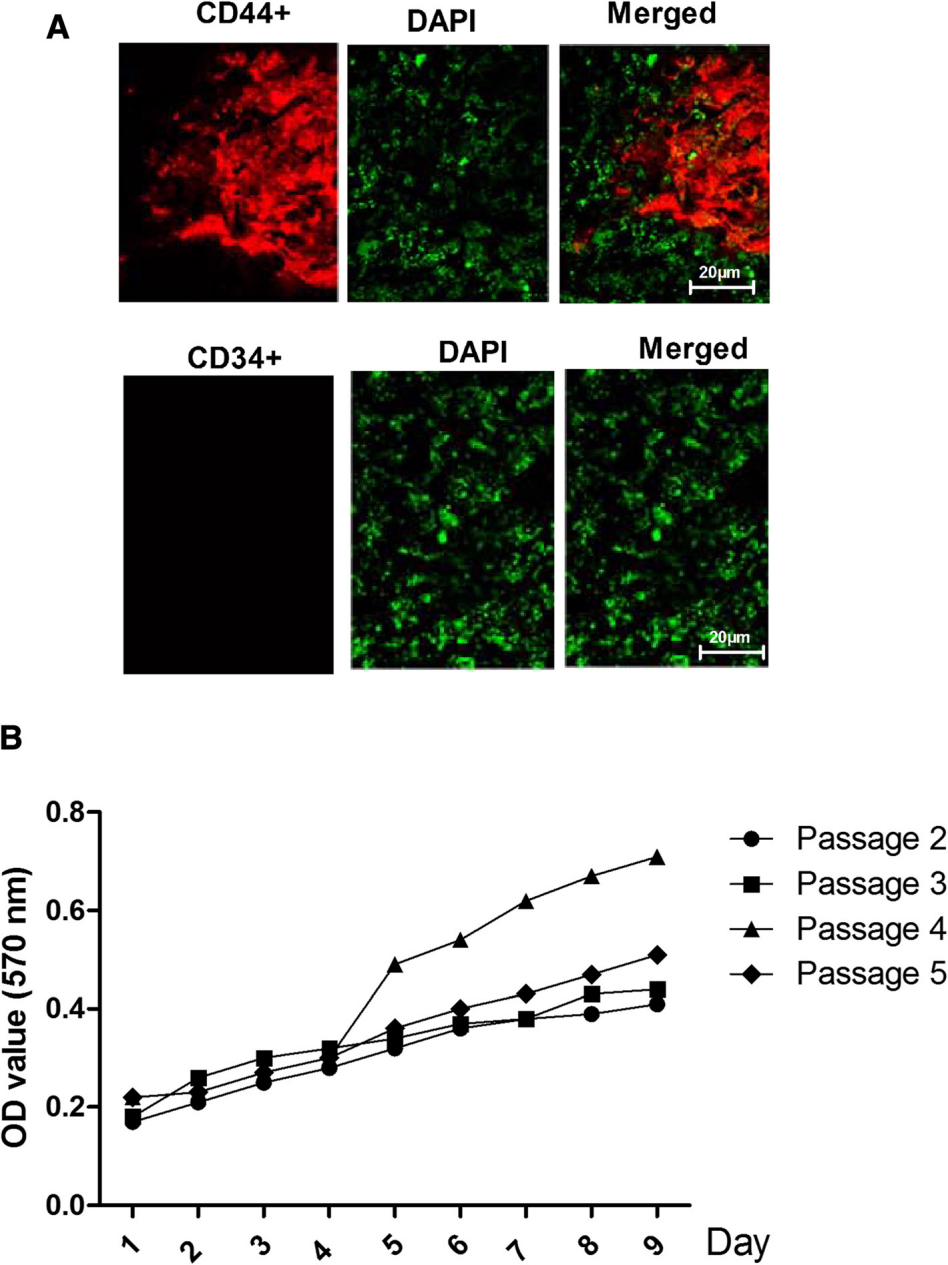
Identification of rBMSC. **a** Immunofluorescence staining showing the expression of CD34 and CD44 in cultured rBMSC. **b** Cellular proliferation during the second through fifth cell passages (as determined at an absorbance of 570 nm)

At day ten, cells reached 80 % confluence. At day 13, the cells displayed a uniform spindle shape and reached 100 % confluence. To further identify the rBMSC, the expression of CD34 and CD44 was examined by immunofluorescence. The cells were CD44 positive but CD34 negative (Fig. 1a).

The proliferation of the cells in the second through the fifth cell passages was analyzed using an automated microplate reader at an absorbance of 570 nm on days 1 to 10 (Fig. 1b). The cells from the fourth and fifth passages were 100 % confluent at seven days and then entered a lag phase. However, the cells from the second and third passages entered the lag phase two days later. Thus, the cells from the fourth passage displayed the greatest ability to proliferate (*P <* 0.05).

### GHSR expression in rBMSC

**Fig. 2:**
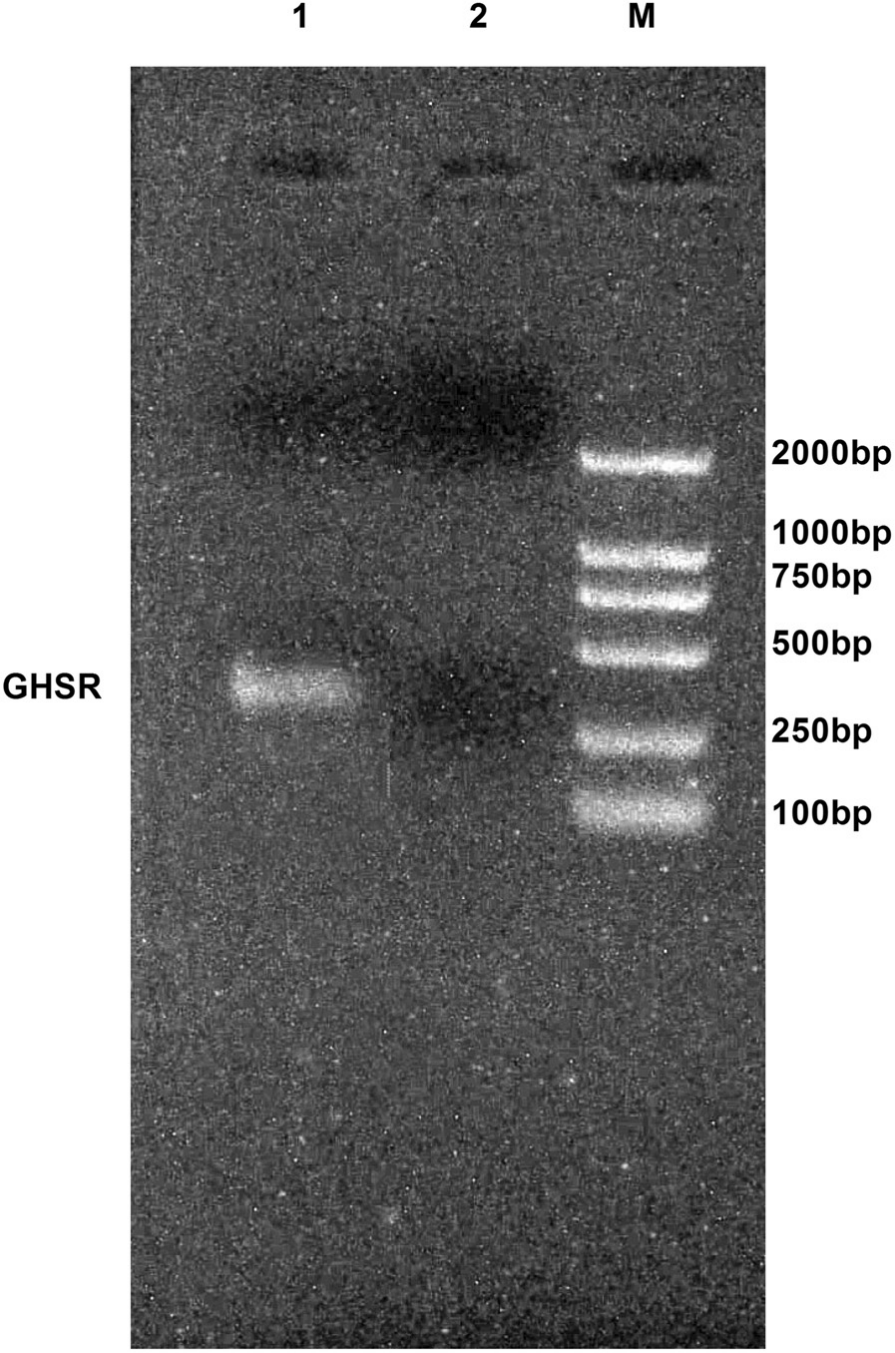
Expression of GHSR mRNA in rBMSCs by RT-PCR

RT-PCR was used To detect the expression level of GHSR mRNA in the rBMSC,. GHSR was expressed at a high level in the rBMSC (Fig. 2).

### Optimal Concentrations and Timing of Ghrelin and D-Lys3-GHRP-6Treatment of rBMSC

The MTT assay was used to assess cell growth and viability following treatment of rBMSC with varying concentrations of ghrelin, and to determine the appropriate duration of treatment to achieve the desired level of growth.

**Fig. 3:**
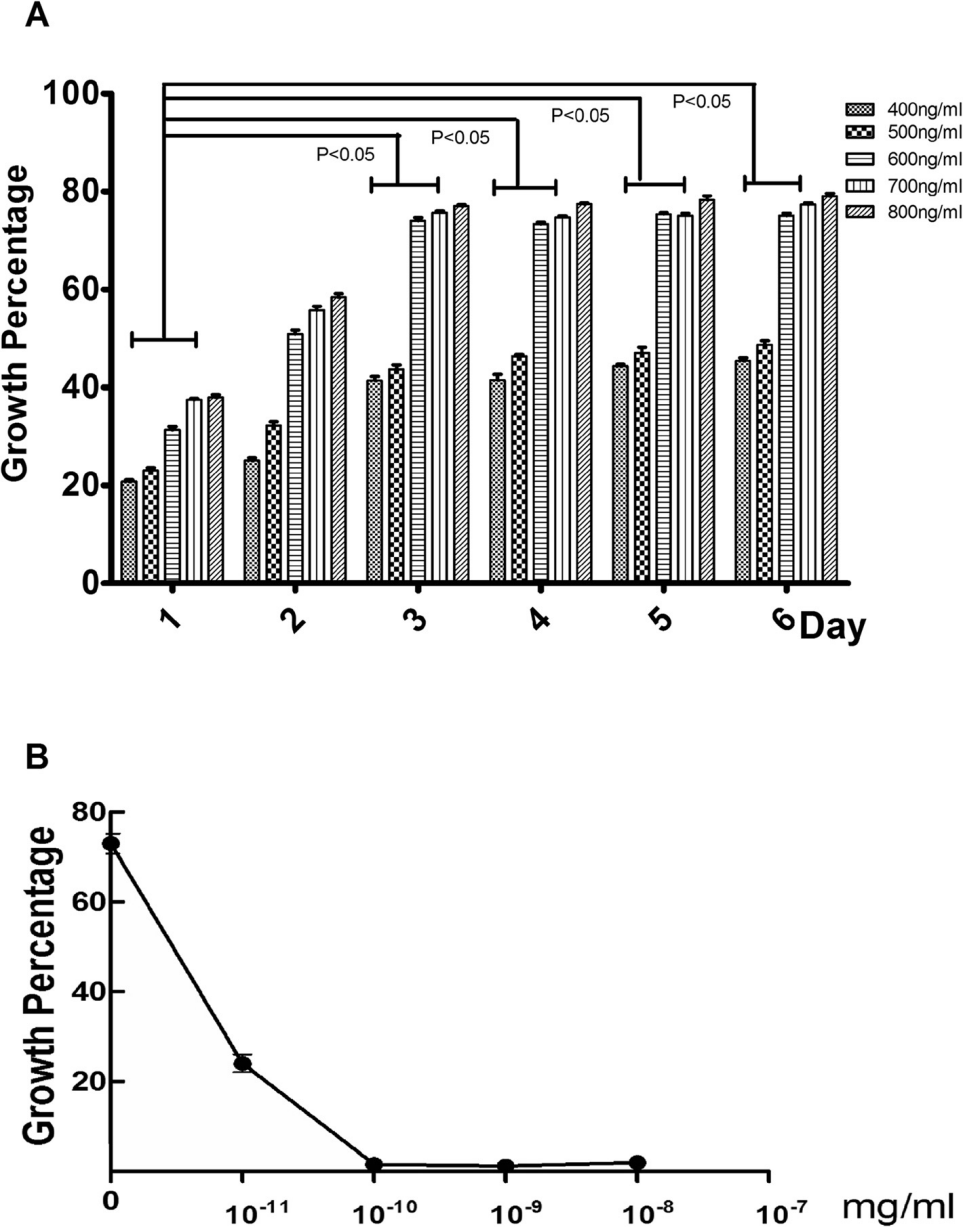
Growth of rBMSCs following treatment with ghrelin and with ghrelin plus D-Lys3-GHRP-6. **a**: Growth percentage at 1 to 6 days following treatment with increasing concentrations (400, 500, 600, 700, and 800 ng/ml) of ghrelin. Presented as mean ± SD (n = 5). **b**: The effect of D-Lys3-GHRP-6 (10^−11^, 10^−10^, 10^−9^, and 10^−8^ mg/ml) treatment on cell growth following ghrelin treatment

Ghrelin was added to the growth medium to final concentrations of 400, 500, 600, 700 and 800 ng/ml. The numbers of viable cells were assessed at 1 to 6 day (Fig. 3a). The results showed that the optimal concentration and duration of ghrelin treatment for rBMSC was 3days of treatment at 600ng/ml ghrelin.

Next, we determined the concentration of D-Lys3-GHRP-6 necessary to promote the growth caused by 600ng/ml ghrelin. rBMSC that had been treated with 600 ng/ml ghrelin were treated with D-Lys3-GHRP-6 at concentrations of 10^−8^, 10^−9^, 10^−10^, and 10^−11^mg/ml, and cell numbers were evaluated at day three (Fig. 3b). The result showed that 10^−10^mg/ml D-Lys3-GHRP-6 could inhibit the growth caused by 600ng/ml ghrelin.

### Ghrelin accelerates the Growth of rBMSC via the ERK1/2 Pathway

**Fig. 4:**
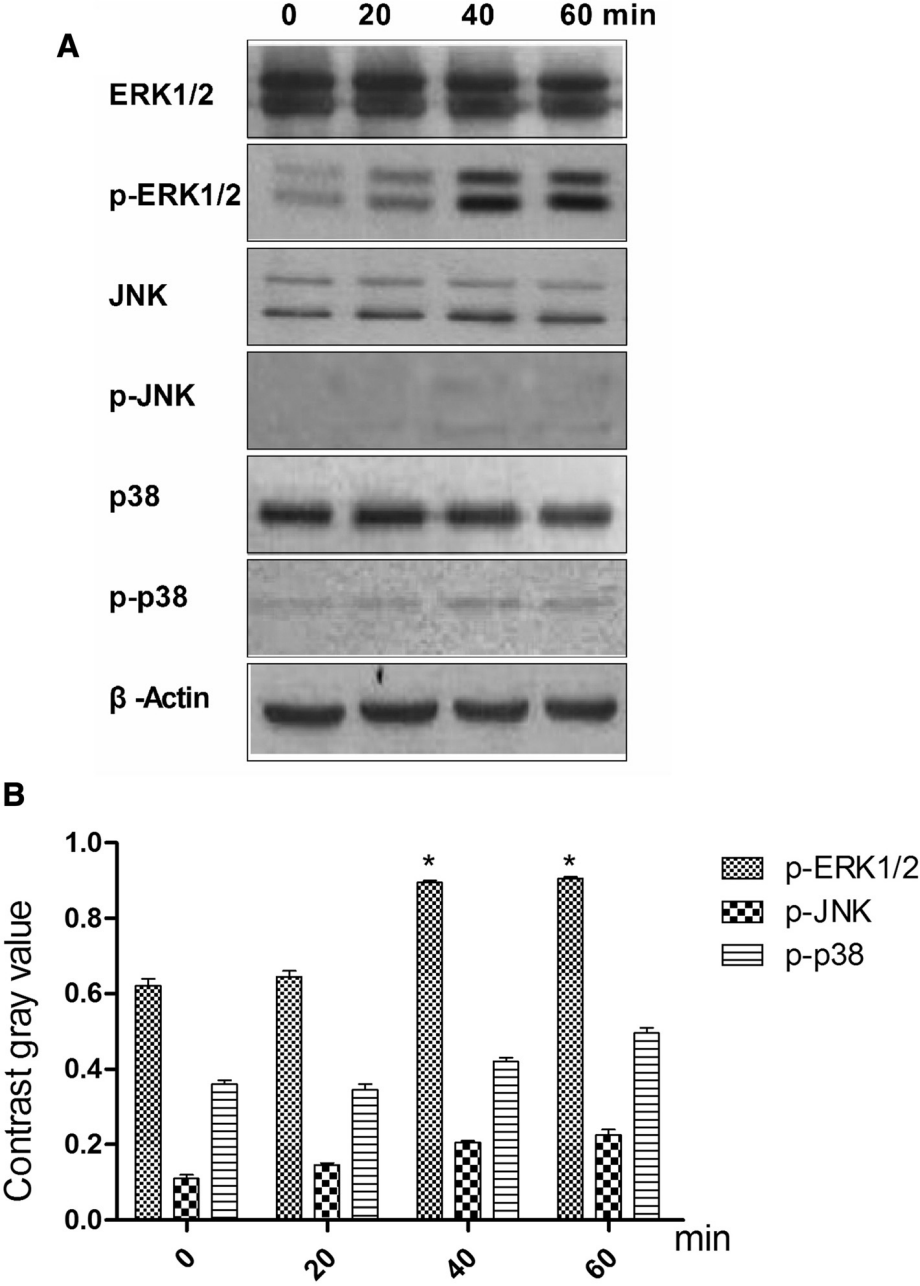
Effects of 600 ng/ml ghrelin on mitogen-activated protein kinase (MAPK) activation in rBMSCs cells. **a**: The expression of total and phosphorylated ERK1/2, JNKs, and p38 proteins. **b**: Contrast gray value of phosphorylation of ERK1/2, JNKs, and p38 based on the western blot. Presented as mean ± SD (n = 5). *Significantly different from the 0 min group (*P <* 0.05)

**Fig. 5:**
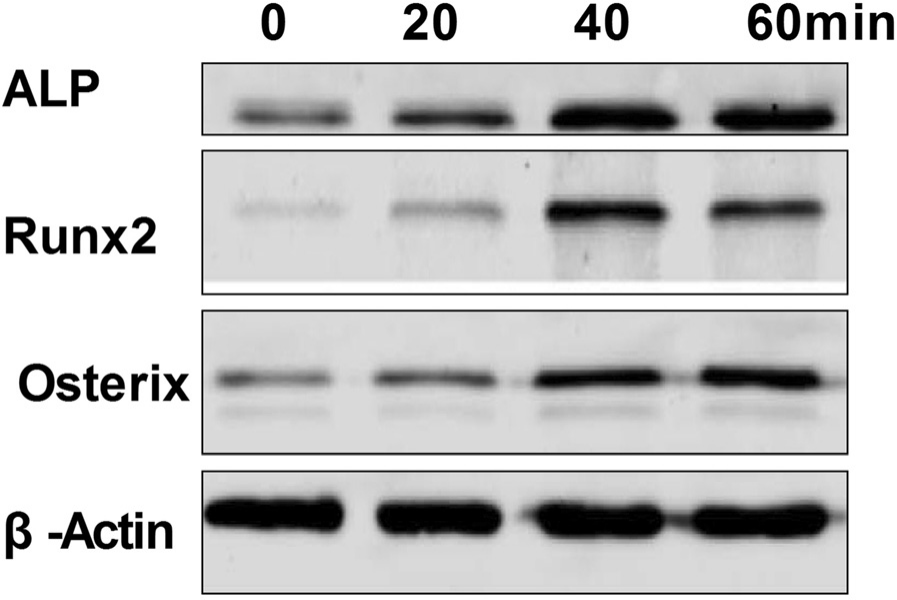
Expression levels of ALP, Runx2, and Osterix proteins in osteoblasts

Phosphorylation states of the MAPKs ERK1/2, JNK, and p38 were detected by their phosphorylation antibody which mainly combinate to the phosphorylation part in these protein. To determine which pathway mediates ghrelin’s acceleration of rBMSC growth, the phosphorylation states of the MAPKs ERK1/2, JNK, and p38 were detected at 0, 20, 40, and 60 min (Fig. 4a) following treatment with 600ng/ml ghrelin. The resulting increase in ERK1/2 phosphorylation was greater than that of JNK or p38 after 40min (Fig. 4b). To explore the function of ghrelin on rBMSC-derived osteoblasts, the rBMSC were allowed to differentiate into osteoblasts, and then treated with ghrelin for 0, 20, 40, and 60 min. The expression levels of ALP, RUNX2, and Osterix were then examined. The expression of these proteins at 40 min was higher than at the other time points (Fig. 5).

**Fig. 6:**
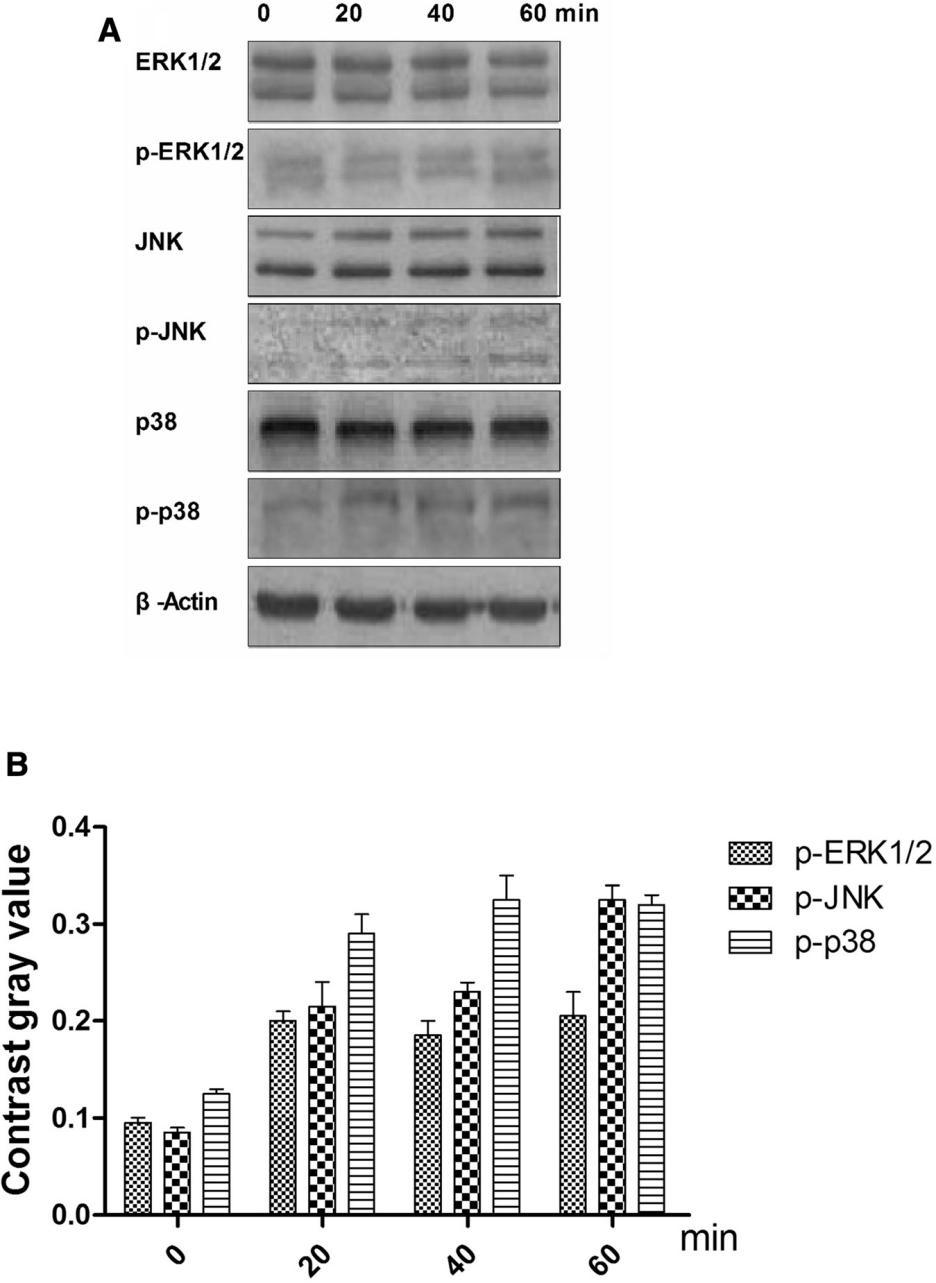
Effects of ghrelin receptor inhibitor (D-Lys3-GHRP-6) on ghrelin-mediated MAPK activation in rBMSC cells. **a**: The expression of total and phosphorylated ERK1/2, JNKs, and p38 proteins. **b**: Contrast gray value of phosphorylation of ERK1/2, JNKs and p38 based on the western blot. Presented as mean ± SD (n = 5). * Significantly different from the 0 min group (*P <* 0.05)

**Fig. 7:**
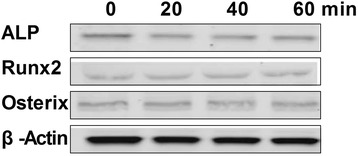
Expression of ALP, Runx2, and Osterix proteins by osteoblasts after treatment with the ghrelin receptor inhibitor (D-Lys3-GHRP-6)

When this treatment was repeated with the addition of 10^−9^mg/ml D-Lys3-GHRP-6, compared with treatment with ghrelin only, the phosphorylation of ERK1/2 was reduced (Fig. 6a). The phosphorylation status of the JNKs and p38 were not significantly changed (Fig. 6b), nor were the levels of ALP, RUNX2, and Osterix (Fig. 7).

**Fig. 8:**
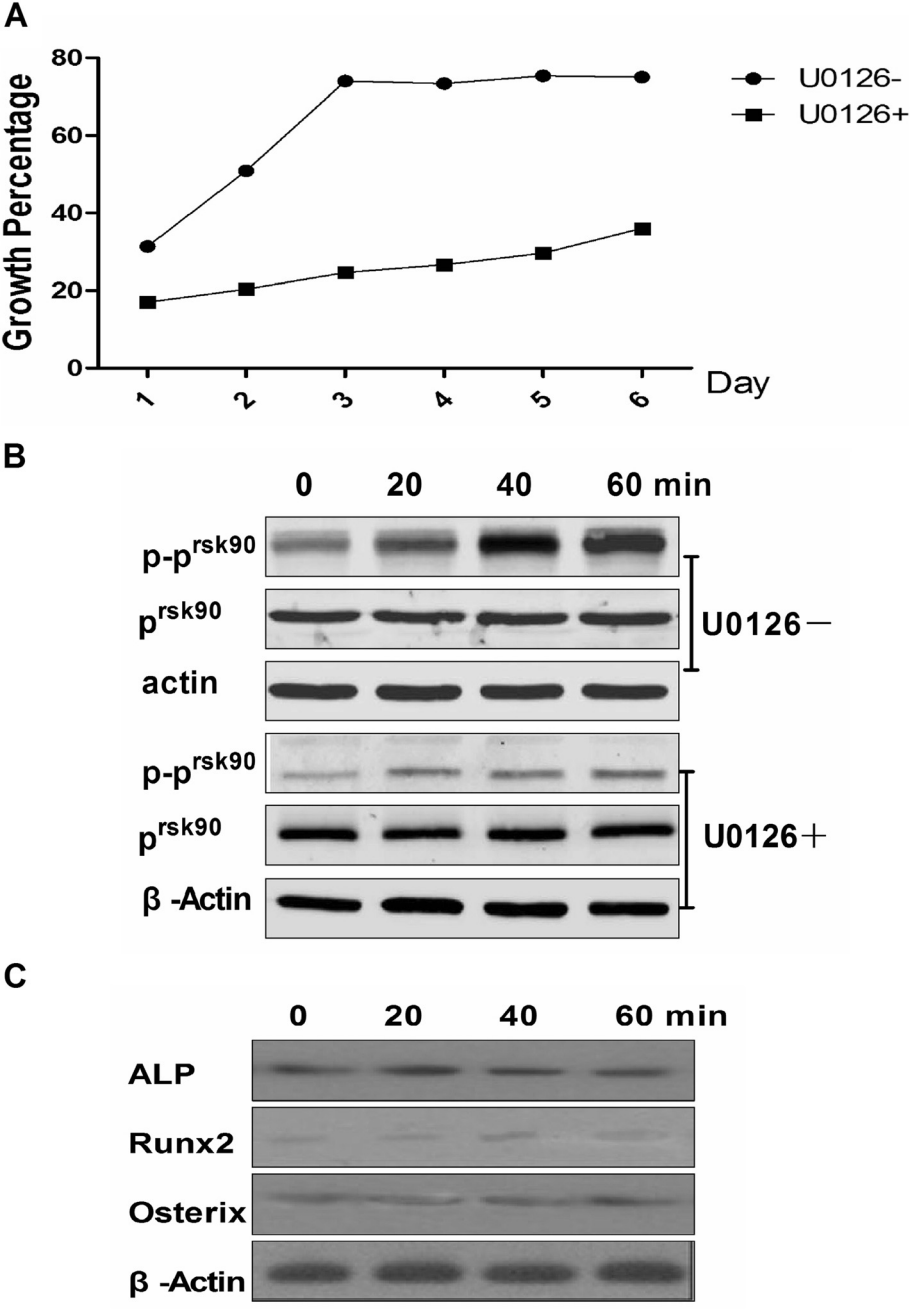
Effect on rBMSCs following treatment with U0126 (a specific inhibitor of ERK1/2 phosphorylation). **a** The growth rate of rBMSCs following treatment with U0126. U0126-: medium plus ghrelin; U0126+: medium plus ghrelin and U0126. **b**: Effects on p^90rsk^ and phosphorylation of p^90rsk^ after the phosphorylation ERK1/2 was inhibited at (0, 20, 40, and 60 min). U0126-: medium plus ghrelin; U0126+: medium plus ghrelin and U0126. **c**: Expression of ALP, Runx2, and Osterix proteins by osteoblasts after the phosphorylation ERK1/2 was inhibited, at 0, 20, 40 and 60 min

These results suggested that ERK1/2 plays a key role in ghrelin’s ability to accelerate the growth of rBMSC. To test this, U0126 (a specific inhibitor of ERK1/2 phosphorylation) was used to silence ERK1/2 expression. In the presence of U0126, ghrelin (600ng/ml) did not accelerate rBMSC growth (Fig. 8a), and the growth rate was significantly lower than in the presence of ghrelin alone. The phosphorylation of p^90rsk^ (p-p^90rsk^) was reduced (Fig. 8b), as were the levels of ALP, RUNX2, and Osterix (Fig. 8c).

## Discussion

Ghrelin is produced mainly by the stomach, although lesser amounts are also produced by the bowel, pancreas, pituitary, kidney, and placenta. The ghrelin receptor, GHSR, is a typical G-protein-coupled seven-pass transmembrane receptor [22]. Several studies have identified the protective effects of ghrelin on the reproductive system [23–26]. Other studies have shown that ghrelin affects the function of embryonic stem cells [27, 28] via the ERK1/2 pathway, but have not focused on rBMSC.

In this study, we verified the identity of rBMSC by their morphology and the finding that they expressed CD44, a marker of rBMSC, but not CD34, a closely related molecule not expressed by rBMSC. we found that ghrelin accelerates the growth of rBMSC through activation of the ERK1/2 branch of the MAPK pathway. Cellular proliferation following the second through fifth passages was analyzed on days 1 to 10 following the passages. The fourth generation rBMSC showed the greatest capacity for proliferation, whereas the second generation showed the least. The optimal ghrelin treatment to obtain maximum growth was 600ng/ml of ghrelin for 3days. We also demonstrated that this effect of ghrelin is mediated through its receptor, GHSR [29], which we showed to be expressed at high levels in rBMSC. Experiments in which the GHSR inhibitor D-Lys3-GHRP-6 blocked the ghrelin-mediated growth provided further support for the role of GHSR.

To gain further insight into the mechanism by which ghrelin accelerate rBMSC growth, we evaluated the activity of signaling pathways downstream of GHSR. The MAPKs are a super-family of serine/threonine kinases that includes ERK, JNK, and p38. These kinases are involved primarily in the activation of nuclear transcription factors that control cell proliferation, differentiation, and apoptosis [30]. Our results suggest that ghrelin accelerates rBMSC growth via the ERK signaling pathway, and not through the activation of JNK or p38. We found that 20 to 60 min of ghrelin treatment was required to stimulate phosphorylation of ERK, and therefore that the stimulus is time-dependent. Furthermore, both blockade of GHSR and ERK by chemical inhibition suppressed the ghrelin-mediated acceleration of rBMSC growth and promoted rBMSC differentiation to osteoblasts.

## Conclusions

Our results provide evidence that the ghrelin/GHSR signaling pathway accelerate rBMSC growth and promotes rBMSC differentiation to osteoblasts mainly through an ERK-dependent pathway. This study only obtained the ghrelin function on rBMSC in vitro, in future will be in vivo. Thus, our findings suggest that ghrelin might be useful in growing large numbers of rBMSC. Further study is necessary before any clinical application is considered.

## Methods

Unless otherwise specified, all chemicals and reagents were purchased from Sigma-Aldrich (St. Louis, MO, USA). Antibodies to IgG, GAPDH, ALP, Runx2, Osterix, CD44, CD34, U0126 (an inhibitor of phospho-ERK1/2) ERK1/2, JNK, p90^rsk^,phospho-ERK1/2, phospho-JNK and phospho-p90^rsk1^(Ser380) were purchased from the Abcam Corporation, USA.

### Isolation and culture of rBMSC

To obtain the rBMSC, the rabbit was used. The femur from a neonatal New Zealand white rabbit was isolated, and the ends of the femur were opened. The bone marrow was flushed from the femur with low glucose Dulbecco’s modified Eagle’s medium (DMEM) using a 1mL syringe. Cells were harvested into a culture dish, suspended using a Pasteur pipette, seeded into a flask containing DMEM and 15 % fetal bovine serum, and cultured in an incubator with 5 % CO_2_ at 37 °C. The medium was replaced every 2days. When cells grew to a confluence of approximately 85 %, they were passaged with 0.25 % trypsin and 0.1 % EDTA (1:2). Cell growth was monitored using an inverted phase contrast microscope (Nikon Co.). The animal experimental protocols were approved by the Chongqing medical university experimental animal management committee.

### RNA Extraction and RT-PCR

To detected the GHSR expression status in the rBMSC, the RT-PCR was used. Total RNA was isolated from o cells using the RNeasy kit (Qiagen, Hilden, Germany). All RNA samples were treated with RNase-free DNase I to remove genomic DNA contamination. The RNA content of samples was too low to be accurately quantified by spectrometry, and thus, 6.5-μL RNA aliquots were converted to cDNA by reverse transcription, then amplified (TaKaRa, Inc., Dalian, China). The ghrelin receptor PCR primers were: sense, 5’-TCTTCCTTCCTGTCTTCTGTC-3’;antisense, 5’-AGTCTGAACACTGCCACC-3’and the PCR condition was 95 °C 5 min, 95 °C 30 s 57 °C 30 s 72 °C 30 s 30 cycles, 72 °C 10 min.

### 3-(4,5-Dimethylthiazol-2-yl)-2,5-diphenyltertrazolium bromide (MTT) assay

To determine the cell growth percentage, the MTT assay was operated. Cells were grown in 96-well plates (1 × 10^3^ cells/well) supplemented with MGF. Control cells were switched from RPMI-1640 to DMEM containing 0.1 % dimethyl sulfoxide (DMSO). At 1 to 6 days following ghrelin treatment (400, 500, 600, 700 and 800ng/ml ghrelin), 20μL of MTT was added to each well to a final concentration of 0.5 %. After a 4h incubation at 37 °C in the dark, 150μL DMSO was added to each well for 10 min to dissolve the formazan crystals. The absorbance was measured using a microplate reader (EXL800, Cole-Parmer, Vernon Hills, IL, USA) at 490nm. All experiments were repeated three times. The viability of the MGF treated cells was expressed as percentage of population growth plus the standard error of the mean (SEM) relative to that of untransfected control cells. Cell growth was calculated as follows:

$$ \%\  growth = \left( mean\  \exp erimental\  absorbance\hbox{--} mean\  control\  absorbance\right)\ / mean\  control\  absorbance \times 100 $$

### Immunofluorescence

To detected the CD44 and CD34 expressin status, the immunofluorescence was used. The rBMSC were fixed in 3.7 % paraformaldehyde for 30 min at room temperature, permeabilized with 0.5 % Triton X-100 in PBS for 15 min, and blocked with 1 % BSA in phosphate-buffered saline (PBS) with 10 % goat serum overnight at 4 °C. The samples were then stained with primary antibodies diluted in PBS. The primary antibody binding was detected with an Alexa Fluor 488 goat anti-rabbit IgG (H + L) secondary antibody. Images were captured with a Nikon A1 confocal microscope. Experiments were performed in triplicate.

### Western blotting

The detected the protein in MAPK pathway and osteogenic, the western blot was used. The protein homogenates from rBMSC were separated using electrophoresis on 8–12 % sodium dodecyl sulphate/polyacrylamide gels and transferred to nitrocellulose membranes. Membranes were blocked for 30 min at room temperature in PBS buffer containing 5 % fat-free milk and 0.1 % Tween 20. Membranes were then incubated with primary antibody for at least 1 h at room temperature or overnight at 4 °C. The membranes were subsequently washed three times with PBS containing 0.1 % Tween 20, incubated with peroxidase-conjugated secondary antibodies, and developed using ECL reagents (Pierce, Rockford, IL, USA).

### Osteogenic differentiation

To detect the rBMSC differentiation to osteogenic, this experiments was operated. The rBMSC were plated at a density of 5000 cells/cm^2^ and exposed to standard differentiation-inducing media for 21 days. The medium was changed twice per week. Osteogenic differentiation was achieved following standard in vitro protocols. Endothelial differentiation was stimulated by culturing the cells in endothelial growth medium-2 (EGM-2) [31].

### Statistical analysis

Statistically significant differences between gene expression levels were determined using one-way analysis of variance (ANOVA) followed by a Newman–Keuls test with GraphPad Prism version five software (GraphPad Software, La Jolla, CA, USA, www.graphpad.com/company/). Replicates were included in the statistical model. Differences were considered statistically significant at the 95 % confidence level (*P <* 0.05). Data are presented as mean ± S.D.

## Abbreviations

MSCs: Mesenchymal stem cells
rBMSC: Rabbit MSCs
